# Preparation and Characterization of DOPO-ITA Modified Ethyl Cellulose and Its Application in Phenolic Foams

**DOI:** 10.3390/polym10101049

**Published:** 2018-09-20

**Authors:** Yufeng Ma, Xuanang Gong, Chuhao Liao, Xiang Geng, Chunpeng Wang, Fuxiang Chu

**Affiliations:** 1 College of Materials Science and Engineering, Nanjing Forestry University, Nanjing 210037, China; gongxuanang2018@126.com (X.G.); liangchuhao@126.com (C.L.); xiang.19930101@163.com (X.G.); 2 Institute of Chemical Industry of Forestry Products, CAF, Nanjing 210042, China; wangcpg@163.com; 3 Chinese Academy of Forestry, Beijing 100091, China; chufuxiang@caf.ac.cn

**Keywords:** DOPO, itaconic acid, ethyl cellulose, phenolic foams, composites

## Abstract

In order to improve the performance of phenolic foam, an additive compound of 9,10-dihydro-9-oxa-10-phosphaphenanthrene-10-oxide (DOPO) and Itaconic acid (ITA) were attached on the backbone of ethyl cellulose (EC) and obtained DOPO-ITA modified EC (DIMEC), which was used to modify phenolic resin and composite phenolic foams (CPFs). The structures of DOPO-ITA were verified by Fourier transform infrared spectroscopy (FT-IR) and nuclear magnetic resonance (^1^H NMR). The molecular structure and microstructure were characterized by FT-IR spectra and SEM, respectively. Compared with EC, the crystallinity of DIMEC was dramatically decreased, and the diffraction peak positions were basically unchanged. Additionally, thermal stability was decreased and *T*_i_ decreased by 24 °C. The residual carbon (600 °C) was increased by 25.7%. With the dosage of DIMEC/P increased, the *E_a_* values of DIMEC composite phenolic resins were increased gradually. The reaction orders were all non-integers. Compared with PF, the mechanical properties, flame retardancy, and the residual carbon (800 °C) of CPFs were increased. The cell size of CPFs was less and the cell distribution was relatively regular. By comprehensive analysis, the suitable dosage of DIMEC/P was no more than 15%.

## 1. Introduction

Phenolic foam (PF), one of the best thermal insulation materials, offers excellent flame retardant properties, as well as low smoke and low toxicity, and is widely used in aviation, construction, industrial pipelines, and transportation [1,2,3]. Nevertheless, large-scale promotion and application are greatly restricted because of its fragility [3,4,5]. In order to reduce the fragility of PF, the toughening modification of PF is imperative. During the preparation process of PF, petroleum based products (such as glass fibers, aramid fibers) are introduced in PF to improve the toughness of PF [6,7,8] on the one hand, and on the other hand, long and flexible molecular chains (such as polyurethane prepolymer [9], epoxy [3], cardanol [10], etc.) are introduced into the molecular structure of PF to reduce fragility. However, there are few reports about toughening modification of phenolic foam using renewable cellulose.

Cellulose is one of the most abundant, renewable, and environmentally friendly natural macromolecular resources, which offers low price and density, high specific strength, degradability, and non-toxicity. Cellulose has become one of the most concerned polymer reinforcing materials [11,12,13,14]. However, due to its supramolecular structure, cellulose cannot dissolve in water or most organic solvents, which greatly restricts the modification of cellulose [15]. As a cellulose derivative, cellulose ethers have been proved to be particularly useful as intermediates [15]. Ethyl cellulose (EC) is a kind of cellulose ether that has been widely used as biomedical or intelligent materials due to its nontoxicity, biocompatibility, and high mechanical strength [16,17,18,19,20,21]. Since EC is not a flame retardant material, the use of flame retardant to modify EC is necessary with the aim to improve the mechanical properties of the composites without reducing its flame retardancy,

9,10-dihydro-9-oxa-10-phosphaphenanthrene-10-oxide (DOPO) is an excellent flame retardant, and mainly exerts the fire retardant quenching effect by releasing free PO radicals and terminating the chain reaction of combustion in gas phase [22]. The DOPO contains very active phosphor hydrogen bonds, which are prone to result in the reaction of nucleophilic addition. Therefore DOPO has attracted extensive attention in the field of flame retardant modified polymers [23,24,25,26,27,28]. Itaconic acid (ITA) is an important renewable unsaturated dicarboxylic acid and is produced via fermentation with starch. ITA consists of one unsaturated bond and two carboxy functionalities. The conjugacy relation between an unsaturated bond and one carboxy endows ITA with a strong reaction capacity. Therefore, ITA has been used to synthesize polymers by addition, esterification, or polymerization reactions, and has widely utilized in the production of synthetic fibers, resins, adhesives, etc. [29,30,31,32].

Herein, this work aims to introduce a phosphorus compound into the structure of ITA, followed by the modification of EC. Sequentially, the composite PF is prepared from using the modified EC. DOPO and ITA are chosen for their versatilities in organic synthesis [30,31,32,33,34]. It was hypothesized that it could not only improve the mechanical properties of composite PF, but also without reducing the flame retardancy. The structure of DOPO-ITA was characterized by Fourier transform infrared spectroscopy (FT-IR) and nuclear magnetic resonance (^1^H NMR) spectroscopy. The properties of DOPO-ITA modified EC were measured including molecular structure, microstructure, crystallinity, and thermal stability. The curing kinetics of DIMEC composite phenolic resin was studied by the differential scanning calorimetry (DSC) at different heating rates. The mechanical and fragile properties, flame resistance, and microstructure of composite PFs were investigated as well.

## 2. Materials and Methods

### 2.1. Materials

Phenol (P > 99%), formaldehyde (37 wt %), calcium oxide (CaO), and sodium hydroxide (NaOH) were obtained from Nanjing Chemical Reagent, Ltd. (Nanjing, China). 9,10-dihydro-9-oxa-10-phosphaphenanthrene-10-oxide (DOPO) was obtained from Shenzhen jinlong chemical technology Co., Ltd. (Shenzhen, China). Itaconic acid (ITA), Deuterium dimethylsulfoxide (*d*_6_-DMSO) and ethyl cellulose (EC) were purchased from Aladdin (Shanghai, China). Polysorbate-80, petroleum ether, and Paraformaldehyde (≥95%) were obtained from Sinopharm group Chemical Reagent Co. Ltd. (Shanghai, China). Mixed acid curing agent were obtained from Institute of Chemical Industry of Forestry Products, Chinese Academy of Forestry (Nanjing, China).

### 2.2. Methods

#### 2.2.1. Synthesis of DOPO-ITA

DOPO (0.13 mol), ITA (0.1 mol), and Xylene (50 mL) were added into a round bottom flask equipped with magnetic stirring. The reaction was performed for 5 h at 125~130 °C under an inert environment (*N*_2_). After the temperature decreased to 100 °C, the vacuum filtration was performed and tetrahydrofuran (50 mL) was added and obtained a crude DOPO-ITA. The wash of crude DOPO-ITA with tetrahydrofuran and vacuum filtration was repeated three times, and obtained the white and purified solid (DOPO-ITA). The final DOPO-ITA was obtained after the dry at 40 °C to a constant weight under vacuum.

#### 2.2.2. Preparation of DOPO-ITA Modified EC (DIMEC)

**Figure 1:**
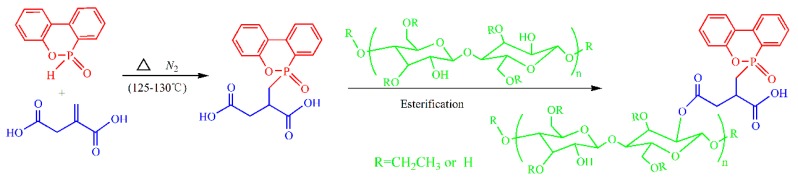
Scheme of DIMEC.

Ethyl cellulose (0.1 mol) and DOPO-ITA (0.05 mol) were added into a round bottom flask equipped with magnetic stirring. Then dimethylformamide (50 mL) and potassium carbonate (0.01 mol) were added. The reaction was performed for 9 h at 120 °C, then the vacuum filtration was performed, and non-reactive materials were removed by extraction. Finally, DIMEC was obtained and dried to a constant weight at 50 °C in a vacuum oven. The yield of DIMEC was about 70.6%. The scheme of DIMEC was shown in Figure 1.

#### 2.2.3. Preparation of Composite PFs

The phenolic resin (PR) was synthesized according to the literature [35]. During the processing of synthesis of PR, DIMEC (5 wt %/P, 10 wt %/P, 15 wt %/P and 20 wt %/P) was introduced in the system of reaction. After the end of the reaction, DIMEC composite PR (DCPR) was obtained. Surfactants (Polysorbate-80, 5%/DCPR), acid curing agents (20%/DCPR) and blowing agents (petroleum ether, 5%/DCPR) were added into the DCPRs and completely mixed, which was then poured into a mold. Phenolic foams were obtained after foaming for 40 min at 70 °C.

### 2.3. Characterizations

FT-IR spectra of DOPO-ITA and DIMEC were monitored by a Fourier transform infrared spectrometer (Nicolet IS10, Madison, WI, USA). ^1^H NMR spectra were performed on a DRX 500 NMR spectrometer (400 MHz) (Bruker, Karlsruhe, Germany) at room temperature using d6-DMSO as solvent, and tetramethylsilane (TMS) as an internal reference. XRD spectra of DIMEC were collected on a Shimadzu 6000× X-ray diffractometer (Kyoto, Japan). SEM were used to observe the micro-scale morphology of DIMEC and PFs by a Hitachi S3400-Nscanning electron microscope (Tokyo, Japan). Thermogravimetric analysis (TGA) curves were collected by a NETZCSH TG 209 F3 TGA system (Bavaria, Germany) under nitrogen atmosphere. Samples were heated from 35 to 600 °C (DIMEC) and 800 °C (CPFs) at a heating rate of 10 °C/min. DSC spectra were obtained on Diamond DSC (PerkinElmer, Waltham, MA, USA). DSC measurements were performed using freeze-dried samples. Heating rates were 5, 10, 15, and 20 °C/min. The scanning temperature ranged from 25 to 200 °C in flowing nitrogen atmosphere (0.02 L/min). Compression strength, bending strength, and tensile strength were measured according to the standard ISO 844:2014, ISO 1209-1:2012, and ISO 1926-2009, respectively. The test was repeated for 5 times. Limiting oxygen indexes (LOIs) of all samples were obtained at room temperature on a JF-3 LOI instrument (LOI Analysis Instrument Company, Jiangning County, China) according to ISO 4589-1-2017, the number of tests was five.

## 3. Results and Discussion

### 3.1. The Properties of DOPO-ITA

#### 3.1.1. FT-IR of DOPO-ITA

**Figure 2:**
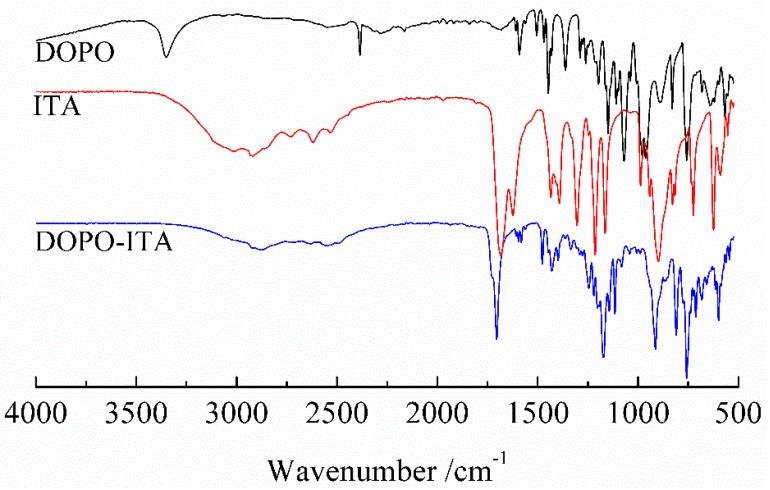
FT-IR of DOPO-ITA.

As shown in Figure 2, the FT-IR analysis of DOPO [36,37,38]: 2385 cm^−1^ (P-H); 1608 cm^−1^, 1591 cm^−1^ and 1557 cm^−1^ (phenyl); 1447 cm^−1^ (P-phenyl); 1260 cm^−1^ (P=O); 892 cm^−1^ (P-O-phenyl). The FT-IR analysis of DOPO-ITA: 1704 cm^−1^ (C=O); 1608 cm^−1^, 1595 cm^−1^ and 1581 cm^−1^ (phenyl); 1429 cm^−1^ (P-phenyl), 1245 cm^−1^ (P=O), 913 cm^−1^ (P-O-phenyl). The FT-IR analysis of ITA [39]: 1682 cm^−1^ (C=O); 1623 cm^−1^ (C=C). Compared with the spectrum of DOPO, the characteristic peak of P-H (in DOPO-ITA) disappeared at 2385 cm^−1^. By comparison with the spectrum of ITA, the characteristic peak of C=C (in DOPO-ITA) disappeared at 1623 cm^−1^. Several new characteristic peaks were observed: 1608 cm^−1^, 1595 cm^−1^ and 1581 cm^−1^ (phenyl); 1429 cm^−1^ (P-phenyl), 1245 cm^−1^ (P=O), 913 cm^−1^ (P-O-phenyl) [40,41,42]. From the FT-IR analysis, it was evident that DOPO-ITA was successfully synthesized.

#### 3.1.2. ^1^H NMR of DOPO-ITA

**Figure 3:**
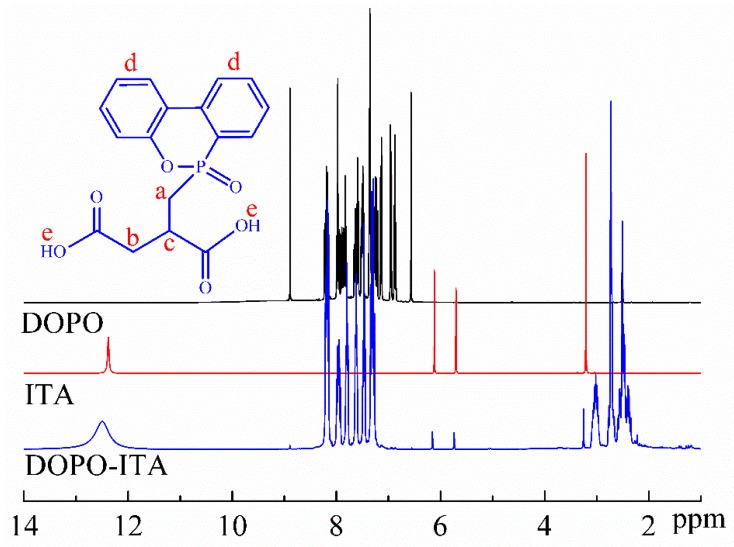
^1^H NMR spectra of DOPO-ITA.

For further confirmation of molecular structure, ^1^H NMR spectrum of DOPO-ITA was recorded and shown in Figure 3. For DOPO, the signal around 6.56–8.89 ppm corresponded to the phenyl protons. For ITA, the signal around 12.45 ppm corresponded to carboxyl protons. For DOPO-ITA, the chemical shifts of H^a^, H^b^, H^c^, H^d^ and H^e^ were observed at 2.41 ppm, 2.72 ppm, 3.26 ppm, 7.27–8.21 ppm, and 12.45 ppm, respectively [40,41,42,43]. The protons of phenyl group (7.26–8.22 ppm) and carboxyl protons (12.45 ppm) appeared in the spectrum of DOPO-ITA. These results support that the reaction occurred between DOPO and ITA.

### 3.2. The Properties of DOPO-ITA Modified EC (DIMEC)

#### 3.2.1. FT-IR of DIMEC

**Figure 4:**
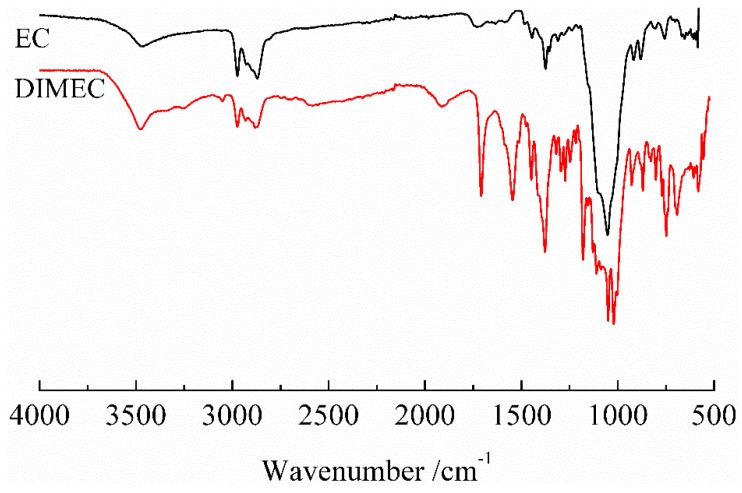
FT-IR of DIMEC.

As shown in Figure 4, a strong peak at 1708 cm^−1^, corresponding to C=O stretching vibration of the ester bond, is observed. The peaks at 1548 cm^−1^ and 1448 cm^−1^ correspond to benzene ring. A peak at 1246 cm^−1^ corresponds to P=O stretching. A peak at 1180 cm^−1^, corresponding to C-O stretching vibration of the ester bond, and a peak at 927 cm^−1^ representing P-O-Ph stretching, are clearly observed. The appearance of new peaks confirmed that the reaction occurred between DOPO-ITA and EC, DOPO-ITA was introduced in the molecular structure of EC.

#### 3.2.2. XRD of DIMEC

**Figure 5:**
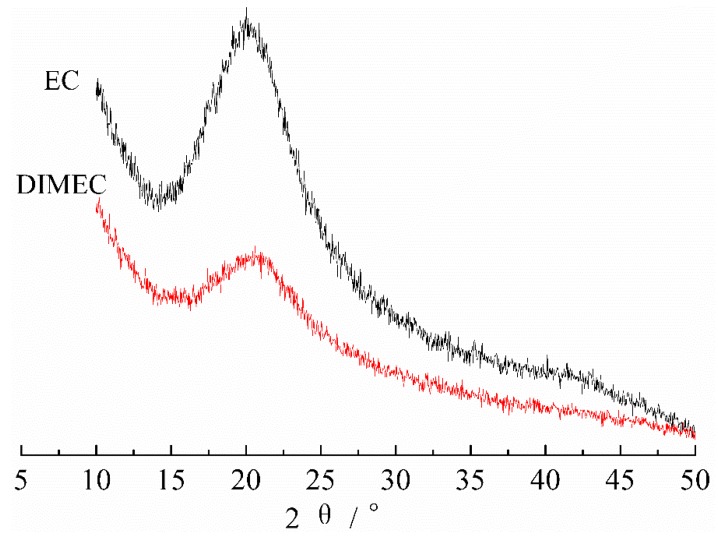
XRD of DIMEC and EC.

Figure 5 shows the XRD spectra of DIMEC and EC. The peaks appeared in 2θ = 19.98° [44]. Compared with EC, the peak positions of DIMEC were basically unchanged, despite the significant attenuation of the peak intensity. The result indicated that the crystal structure of DIMEC was not destroyed. It could be explained by the fact that the surface of modified EC was covered by DOPO-ITA, which led to the significant decrease of the crystallinity of DIMEC.

#### 3.2.3. SEM Micrographs of DIMEC

**Figure 6:**
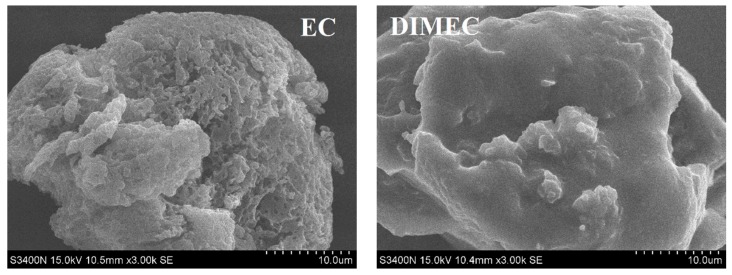
SEM micrographs of DIMEC and EC.

SEM micrographs (3000×) of DIMEC and EC are shown in Figure 6. The surface of EC was very rough, and has lots of holes. Compared with EC, the surface of DIMEC was smoother and covered by a thin layer of material. This might be due to the fact that during the process of modification, the esterification reaction occurred between DOPO-ITA and EC. DOPO-ITA was introduced in the molecular structure of EC. Finally, the surface of EC was covered by DOPO-ITA.

#### 3.2.4. TG and DTG of DIMEC and EC

**Figure 7:**
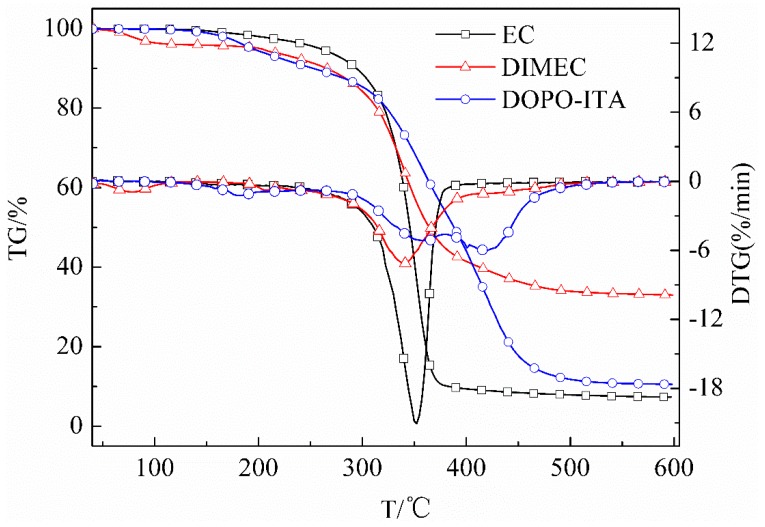
TG and DTG of DIMEC and EC.

Figure 7 shows TG and DTG of DIMEC and EC XCD. The initial decomposition temperatures (*T*_i_) [45] of EC and DIMEC were 322.8 °C and 298.8 °C, respectively, and the carbon residues (600 °C) were 7.29% and 32.99%, respectively. It was observed that *T*_i_ of DIMEC was less than that of EC, but the carbon residue (600 °C) of DIMEC was more than that of EC. It could be explained that *T*_i_ (152.9 °C) of DOPO-ITA was less than that of EC, and therefore, there was no positive significance to improve the heat resistance of DIMEC. However, the carbon residue (600 °C) (10.51%) of DOPO-ITA was more than that of EC, otherwise, biphenyls heterocycle was introduced in the molecular structure of DIMEC by modification, and led to the increase of the carbon content of DIMEC. Thus, the carbon residue (600 °C) of DIMEC was remarkably improved.

### 3.3. The Curing Kinetics of DCPRs

**Figure 8:**
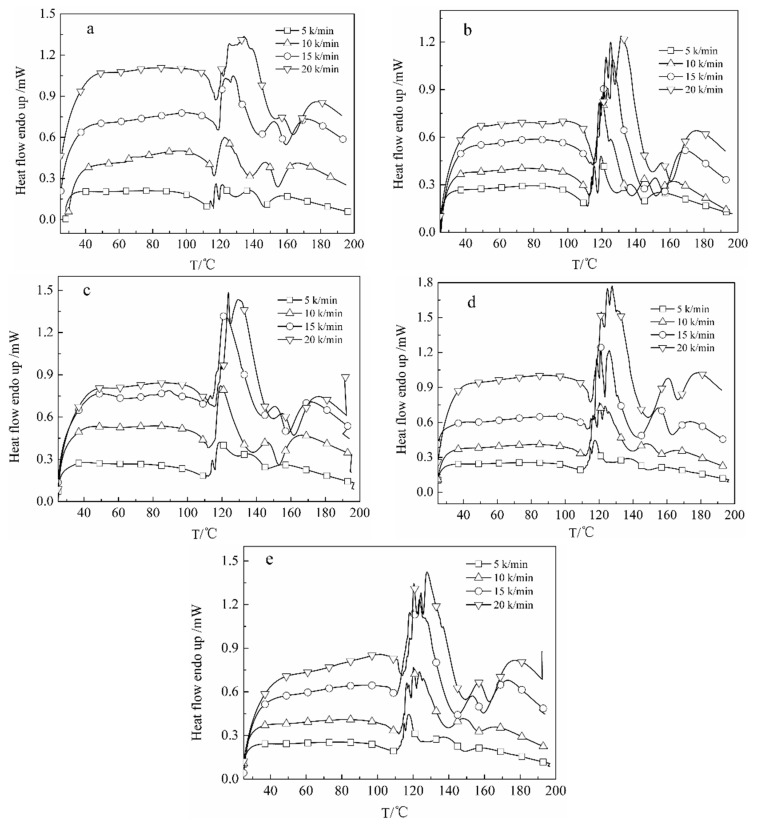
DSC curves of DCPRs ((**a**) 0%; (**b**) 5%; (**c**) 10%; (**d**) 15%; (**e**) 20%).

**Figure 9:**
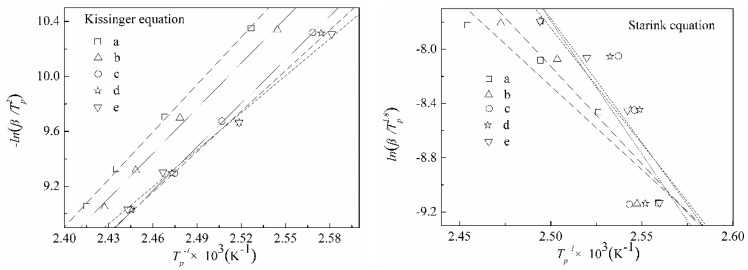
Linear fitting charts of −ln(*β/T_p_*^2^) and −ln(*β/T_p_*^1.8^) versus 1/*T_p_* ((**a**) 0%; (**b**) 5%; (**c**) 10%; (**d**) 15%; (**e**) 20%).

**Figure 10:**
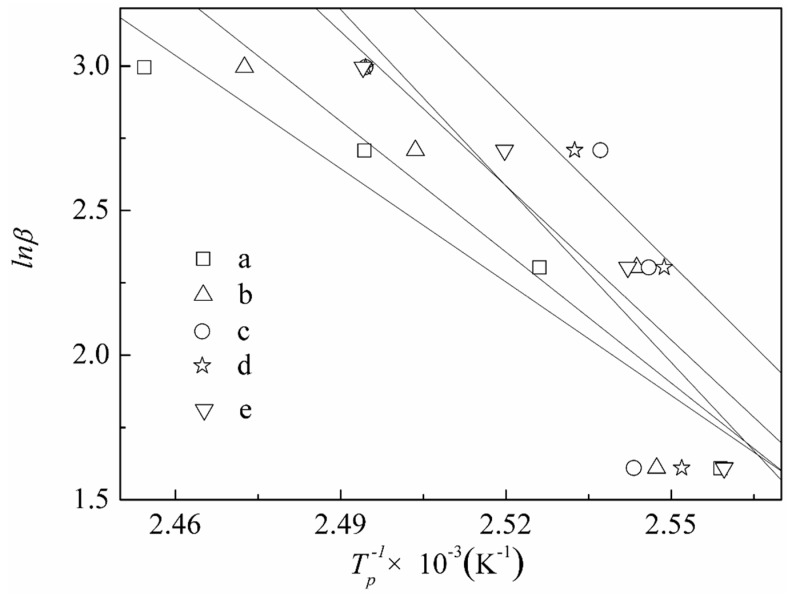
Linear fitting charts of ln*β* versus 1/*T_p_* ((**a**) 0%; (**b**) 5%; (**c**) 10%; (**d**) 15%; (**e**) 20%).

The Kissinger [46] and Ozawa [47] isoconversion methods were applied for the obtaining activation energies of *E_a_*. It is shown that from these two methods, the Kissinger method is generally the most accurate. A new Starink [48] isoconversion method is obtained, which is shown to be significantly more accurate than the others. Values for *E_a_* were obtained according to Kissinger’s equation (Equation (1)) [46] and the Starink equation (Equation (2)) [48]. Where *β* was the heating rate (K/min), *T_p_* was the peak temperature (K), *R* was the gas constant (8.314 J·mol^−1^·K^−1^). DSC curves of DCPRs are shown in Figure 8. Plotting −ln(*β*/*T_p_*^2^) and ln(*β*/*T_p_*^1.8^) versus 1/*T_p_* (Figure 9), should give a straight line of slope, and therefore the activation energies of curing reactions were obtained. The curing reaction orders of high-solid resol phenolic resins were obtained by plotting ln*β* versus 1/*T_p_* (Figure 10), as indicated by the subscripts in the Crane equation (Equation (3)) [49].

(1) $$-\ln(\frac{\beta }{T_{p}^{2}})=-\ln(\frac{AR}{E_{a}})+(\frac{1}{T_{P}})(\frac{E_{a}}{R}).$$

(2) $$\ln(\frac{\beta }{T_{p}^{1.8}})=-1.0037\frac{E_{a}}{RT_{P}}+cons\tan t$$

(3) $$\frac{d(\ln\beta )}{d(\frac{1}{T_{p}})}=-\frac{E_{a}}{nR}$$

Table 1 shows the DSC data of DCPRs. The *E_a_* values were basically not change much which calculated by the Kissinger and Starink isoconversion methods. With the increasing of dosage of DIMEC/P, the *E_a_* values of DCPRs were increased gradually. This might be explained that the reactions might be occurred between hydroxyl (in DIMEC) and methylol phenol, whose individual reactions had higher activation energy values [50]. Otherwise, there were the carboxyl groups (in DIMEC), DIMEC was acidic. With the dosage of DIMEC/P increased, the pH values of DCPRs curing system were decreased, which possibly leaded to the increase of the *E_a_* values of DCPRs cure [50,51]. And the reaction orders were all non-integers, this showed that the curing reaction were quite complicated.

### 3.4. The Properties of Composite PFs (CPFs)

#### 3.4.1. Compression and Bending Strength

**Figure 11:**
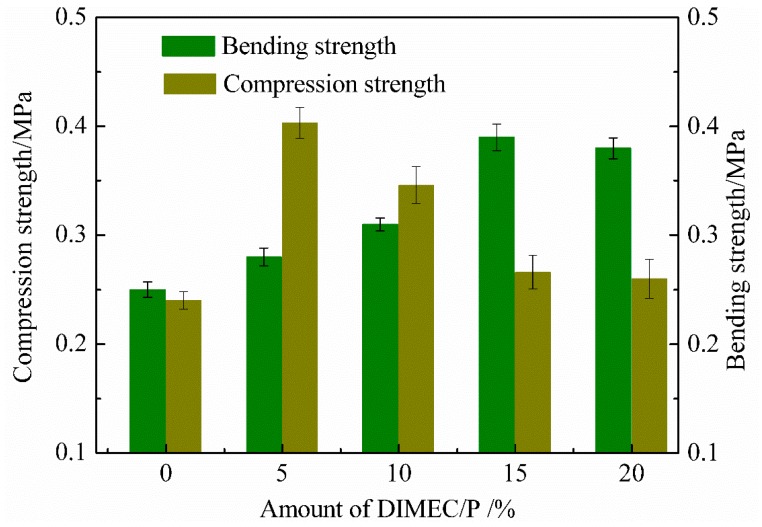
Compression and bending strength of CPFs.

Compression and bending strength of CPFs is shown in Figure 11. With the increase of the dosage of DIMEC/P, the compression strength of CPFs gradually decreased, whereas the bending strength of CPFs gradually increased. Nevertheless, the amount of DIMEC/P was more than 15%, and the bending strength of CPFs was slightly decreased. The compression strength was almost unchanged and the compression and bending strength were more than those of PF. It could be explained by the fact that DIMEC was introduced into CPFs, and that the toughness of CPFs was improved. Therefore, the capacity of resistance compression was reduced and the ability of resistance bending was increased and significantly better than PF. However, when the dosage of DIMEC was higher, the cell structures of CPFs were destroyed and the mechanical properties of CPFs were deteriorated. Therefore the suitable dosage of DIMEC/P was no more than 15%.

#### 3.4.2. Tensile Strength

**Figure 12:**
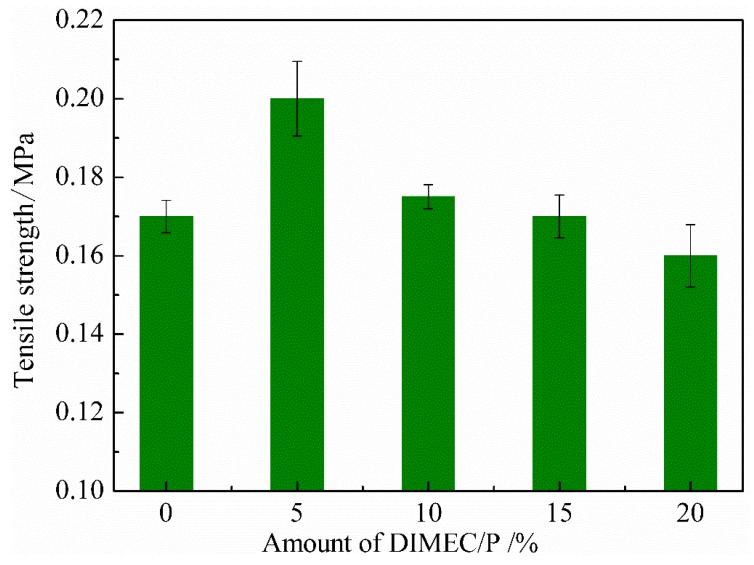
Tensile strength of CPFs.

As shown in Figure 12, the tensile strength of CPFs was decreased with the increase of the amount of DIMEC/P. When the dosage of DIMEC/P was 15%, the tensile strength of CPFs was the same as that of PF, and was then less than that of PF. This phenomenon was due to the fact that when DIMEC was introduced into CPFs, the toughness of CPFs was improved along with the destruction of the cell structures of CPFs l. There should be a balance between toughening and breaking. When the amount of DIMEC/P was less (≤15%), toughening was in the ascendant compared with breaking. Therefore the tensile strength of CPFs was more than or equal to that of PF. And then breaking was in the ascendant, the tensile strength of CPFs was less than that of PF. Thus, the suitable dosage of DIMEC/P was no more than 15%.

#### 3.4.3. Fragility

**Figure 13:**
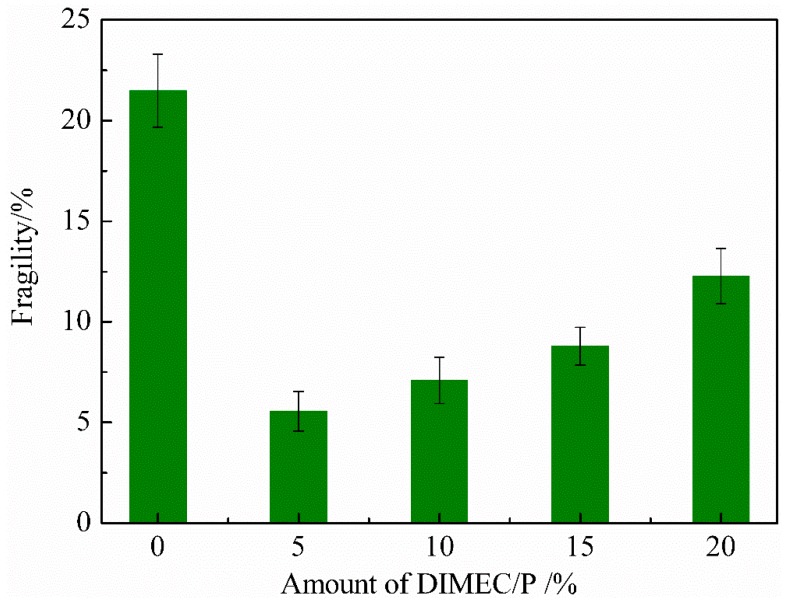
Fragility of CPFs.

The ratio of mass loss is often used to characterize the fragility of foams. The more fragile the foam is, the greater the ratio of mass loss is. Figure 13 shows the fragility of CPFs. The analysis revealed that the ratios of mass loss of CPFs were increased with the increase of dosage of DIMEC/P. When the dosage of DIMEC/P was less than or equal to 15%, the ratio of mass loss of CPFs was less than 8.79%, and the ratio of mass loss of CPFs was 12.28%. However the ratios of mass loss of CPFs were less than that of PF. The reason could be explained by the fact that with the increasing dosage of DIMEC, the original bubble structure was destroyed and the bubble uniformity was decreased, which led to the loss of mass increased. Otherwise, the toughness of CPFs was improved with the introduction of DIMEC. Therefore the ratios of mass loss of CPFs were less than that of PF. The results showed that the dosage of DIMEC/P was not too much, and the better content of DIMEC/P was no more than 15%.

#### 3.4.4. Limited Oxygen Index (LOI)

**Figure 14:**
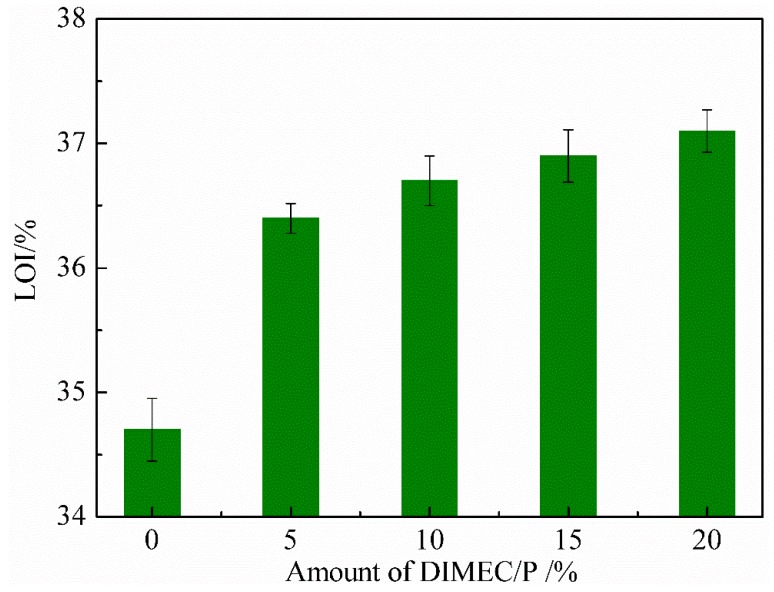
LOI of CPFs.

As shown in Figure 14, LOIs of CPFs were gradually increased with the increase of the addition of DIMEC/P, and were more than that of PF. Nevertheless, the amplitude of variation was modest (36.4–37.1%). And these foams were considered as the flame resistant materials (LOI ≥ 27%) [52]. The results showed that although the variation amplitude of LOI was small, it positively improved the LOIs of CPFs by DIMEC added. This could be explained by the fact that the phosphorus element (in DIMEC) was introduced into CPFs during the process of combustion, the fire retardant quenching effect could be exerted by releasing free PO radicals and terminating the chain reaction of combustion in gas phase [22]. Therefore, LOIs of CPFs were more than that of PF. And with the increasing of DIMEC/P, there were more and more flame retardants introduced into CPFs, and LOIs were improved slightly.

#### 3.4.5. TG and DTG

**Figure 15:**
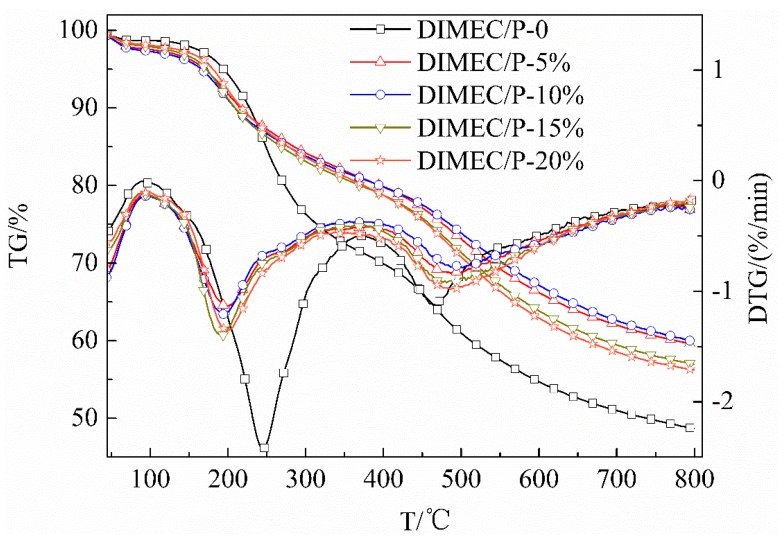
TG and DTG of CPFs.

Figure 15 shows the TG and DTG of CPFs. The initial decomposition temperature (*T*_i_) [45] of PF was 196.1 °C and the carbon residue (800 °C) was 48.67%. With the dosage of DIMEC/P increased, *T*_i_ of CPFs were 158.3 °C, 157.4 °C, 158.8 °C, and 166.6 °C, respectively. The carbon residues (800 °C) were 59.45%, 59.87%, 56.94%, and 56.23%, respectively. Compared with PF, the maximum *T*_i_ was decreased by 38.7 °C, whereas the maximum carbon residue (800 °C) was increased by 11.20%. The result showed that there was no positive significance to improve the heat resistance of CPFs. However, there was a positive significance to improving the heat resistance of CPFs at high temperature. It might be explained by the fact that the reactions occurred among DIMEC, phenol, and formaldehyde and some small molecular compounds were produced. Therefore the heat resistance of CPFs was reduced. Otherwise, phosphorus (in DIMEC) was introduced into the CPF, which could migrate to the external char layer, form a thick and compact thermal barrier, and could delay the process of degradation [53]. So the carbon residues (800 °C) of CPFs were improved.

#### 3.4.6. SEM

**Figure 16:**
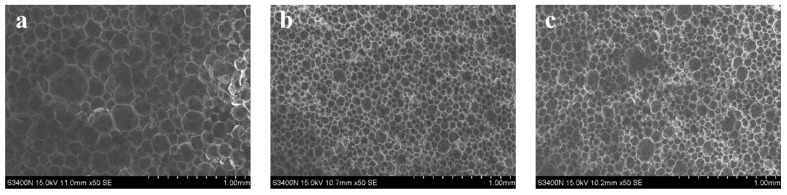

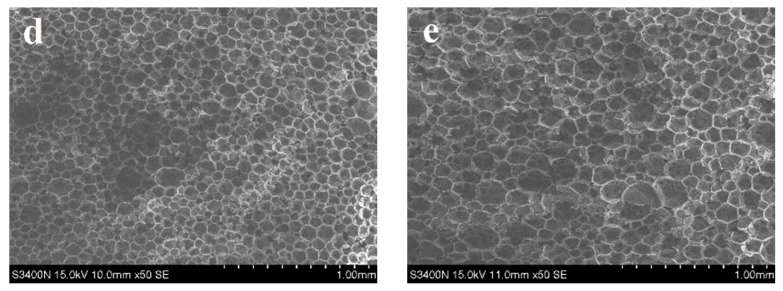
SEM micrographs of CPFs ((**a**) 0%; (**b**) 5%; (**c**) 10%; (**d**) 15%; (**e**) 20%).

SEM micrographs (50×) of CPFs are shown in Figure 16. The cell size of PF was about 100~400 μm. With the addition of DIMEC/P increased, the cell size of CPFs was slightly increased. When the addition of DIMEC/P was less than or equal to 15%, the cell size of CPFs was about 50~100 μm, except a very few large cell sizes were about 150–200 μm. And then the cell size was increased to about at 100~250 μm. The cell sizes of CPFs were less than that of PF. The results showed that when the dosage of DGMWF was less than or equal to 15%, the cell size of CPFs was less. The cell distribution was relatively regular. Therefore the negative influence of DIMEC on the properties of CPFs was less neglected and the mechanical properties of CPFs were better.

## 4. Conclusions

The structure of DOPO-ITA was confirmed by FT-IR and ^1^H NMR spectra. The esterification reaction between DOPO-ITA and EC was verified by FT-IR spectra and SEM, and DOPO-ITA was successfully introduced in the molecular structure of EC. Compared with EC, the crystallinity of DIMEC was dramatically decreased and the diffraction peak positions were basically unchanged. Additionally, thermal stability decreased, but the residual carbon (600 °C) increased significantly. With the dosage of DIMEC/P increased, the *E*_a_ values of DCPRs were increased gradually and the reaction orders were all non-integers. Compared with PF, the mechanical properties, flame retardancy, and the residual carbon (800 °C) of CPFs were increased. The cell size of CPFs was less, and the cell distribution was relatively regular. By comprehensive analysis, the suitable dosage of DIMEC/P was no more than 15%.

## Figures and Tables

**Table 1:** The DSC data of DCPRs.

Content of DIMEC/P	*β* (k·min^−1^)	*T_p_* (°C)	Kissinger Equation [46]		Starink Equation [48]		*n*
			*E_a_* (kJ·mol^−1^)	*r*	*E_a_* (kJ·mol^−1^)	*r*	
0	5	117.64	102.00	0.970	102.66	0.9673	0.94
	10	122.72					
	15	127.76					
	20	134.29					
5%	5	119.42	118.80	08821	119.02	0.8831	0.94
	10	119.98					
	15	126.29					
	20	131.30					
10%	5	120.06	141.28	0.6963	141.41	0.6980	0.95
	10	119.64					
	15	121.00					
	20	127.74					
15%	5	118.72	151.35	0.8215	154.81	0.8787	0.94
	10	119.21					
	15	121.73					
	20	127.74					
20%	5	117.54	162.56	0.9571	162.61	0.9574	0.97
	10	120.23					
	15	123.72					
	20	127.81					
